# The Effect of Temperature on Gonadal Sex Differentiation of Yesso Scallop *Patinopecten yessoensis*

**DOI:** 10.3389/fcell.2021.803046

**Published:** 2022-01-31

**Authors:** Tian Liu, Ruojiao Li, Liangjie Liu, Shaoxuan Wu, Lijing Zhang, Yajuan Li, Huilan Wei, Ya Shu, Yaxin Yang, Shi Wang, Qiang Xing, Lingling Zhang, Zhenmin Bao

**Affiliations:** ^1^ MOE Key Laboratory of Marine Genetics and Breeding, Ocean University of China, Qingdao, China; ^2^ Laboratory for Marine Biology and Biotechnology and Laboratory for Marine Fisheries Science and Food Production Processes, Pilot National Laboratory for Marine Science and Technology (Qingdao), Qingdao, China; ^3^ Laboratory of Tropical Marine Germplasm Resources and Breeding Engineering, Sanya Oceanographic Institution, Ocean University of China, Sanya, China

**Keywords:** temperature, sex differentiation, *FoxL2*, *Dmrt1L*, poikilotherm

## Abstract

Many marine organisms are generally poikilotherms, making seawater temperature one of the most important environmental factors affecting gonadal sex differentiation. Mollusca is the second-largest animal phylum with diverse reproductive systems, but studies on the impact of temperature on sex differentiation are limited to a few sequential hermaphrodites. By combining morphological and molecular analyses, we investigated the effect of temperature on gonadal sex differentiation of a commercially important gonochoristic scallop *Patinopecten yessoensis* in the field and under laboratory conditions. Based on the relative expression of *FoxL2* and *Dmrt1L* in the gonads of 6- to 12 month-old scallops, we found the scallops start to differentiate at 7 months old in September when the seawater temperature was 21°C. To eliminate the effect of factors other than temperature on sex differentiation, we compared the gonadal development of juvenile scallops at different temperatures (21, 16 and 11°C) under laboratory conditions. After 50 days of treatment, the 11°C group contain more germ cell types, and have higher sex differentiation rates than the 21°C group. But no obvious sex bias was observed. These results suggest that high temperature (21°C) inhibits sex differentiation, whereas low temperature (11°C) accelerates sex differentiation by 2 months for this cold-water species. It also supports juvenile *P. yessoensis* is gonochoristic rather than protandrous hermaphroditic. Our study addresses for the first time an environmental influence associated with genetic controls on scallop sex differentiation. It will facilitate a better understanding of how environmental factors affect gonadal development in poikilotherms, especially in the less studied molluscs.

## Introduction

Sexual reproduction commonly exists in the animal kingdom, making sex determination and differentiation one of the most interesting and vital events to investigate. Generally, different genetic or environmental cues can initiate ovarian or testicular pathways, by which an undifferentiated gonad develops to either an ovary or a testis. In mammals, sex is determined by the Y-chromosome gene SRY/Sry, which causes the undifferentiated embryonic gonad to develop as a testis (Nagahama et al., 2021). Unlike mammals, phenotypic sex is easily affected by environmental factors in poikilothermic vertebrates (Miura, 2017; Yu et al., 2021). For example, gonadal sex is determined by the environmental temperature experienced during embryogenesis in the red-eared slider turtle *Trachemys scripta*. Nearly 100% males develop at the male producing temperature of 26°C, and nearly 100% females develop at the female producing temperature of 31°C (Czerwinski et al., 2016). In Nile tilapia *Oreochromis niloticus*, genetic sex determination with a temperature effect has been described. Although the fish displays an XX/XY sex determination system, thermal treatments with temperatures above 32–36.5°C for at least 10 days during the gonadal differentiation period induce masculinization (Nivelle et al., 2019).

Mollusca is the second-largest animal phylum after the Arthropoda, with around 85,000 extant species widespread in marine, freshwater and on land. Molluscs are also the second most important category of aquaculture products after fishes, with global production of 17.5 million tons in 2018 (FAO, 2020). They have a wide diversity of sexual systems, including gonochorism, simultaneous hermaphroditism, and sequential hermaphroditism (Collin, 2013). Like many other poikilothermic animals, the sex and gametogenesis of molluscs are impacted by various environmental factors such as temperature, hormone, culture conditions and pollution (Breton et al., 2018; Adzigbli et al., 2019). For example, a strong male-biased sex ratio was observed at 28°C in spat of Pacific oyster *Crassostrea gigas* (Santerre et al., 2013). In another oyster *Pinctada margaritifera*, females changed to males at higher temperatures and low diet levels, but the gender tended to be maintained if the oysters were fed with high diet levels (Teaniniuraitemoana et al., 2016). In the scallop *Placopecten magellanicus*, sex hormone administration has been reported to shift sex ratios towards males and accelerate gonadal differentiation (Wang and Croll, 2004).

The impact of environmental factors on gonadal development is better understood by recording the expression profile of sex differentiation genes during treatments. In the red-eared slider turtle, temperature-dependent sex determination has been demonstrated to be induced by sexually dimorphic expression of Dmrt1 and its regulator KDM6B (Ge et al., 2017; Ge et al., 2018). Steroid hormones-induced sex reversal in Nile tilapia was demonstrated to be mediated by two important male pathway genes (*gsdf* and *dmrt1*) and two important female pathway genes (*foxl2* and *cyp19a1a*) (Nagahama et al., 2004). In contrast, limited studies have been conducted in molluscs to illustrate the effect of environmental factors on sexual pathway, and most of them focus on protandrous hermaphrodite oysters. For example, the expression of five sex-related genes was investigated to assess the influence of temperature on sex differentiation of oyster *C. gigas*, which suggests involvement of Foxl2 in first gonadic differentiation (Santerre et al., 2013). To evaluate the effect of temperature, food availability and estradiol on the gender of pearl oyster *P. margaritifera*, Teaniniuraitemoana et al. (2016) investigated mRNA expressions of nine genes of the sexual pathway and proposed a probable dominance of genetic sex determinism involving foxl2 and fem1-like in adult oysters.

The Yesso scallop *Patinopecten yessoensis* is a commercially important species widely distributed in China, Japan, Russia and Korea. Adult scallops are predominantly gonochoristic, with scarce hermaphroditism. However, there is controversy over whether juveniles are gonochoristic (Li et al., 2018) or protandrous hermaphroditic (Mori et al., 1977; Maru, 1978; Otani et al., 2017). Considering that temperature is the most important abiotic factor controlling the metabolism of poikilotherms (Nivelle et al., 2019), we investigated the effect of temperature on gonadal sex differentiation in juvenile scallops. Previous study by Li et al. (2018) has shown that *FoxL2* and *Dmrt1L* display sexually dimorphic expression throughout the reproductive cycle, and LOG10 (*Dmrt1L*/*FoxL2*) has been applied to determine the timing of molecular sex differentiation in Yesso scallop. In present study, we first examined the expression of the two early sex marker genes *FoxL2* and *Dmrt1L* in the gonad of 6–12 month-old scallops collected in 2018–2019, and determined the temperature at which sex differentiation occurs. Subsequently, the gonadal development of *P. yessoensis* was investigated by morphological and molecular analysis at three temperatures (21, 16 and 11°C) under laboratory conditions. Our study suggests an influence of temperature on sex differentiation as well as a sexual system of gonochorism rather than protandrous hermaphroditism in juvenile scallops.

## Materials and Methods

### Sample Collection and Temperature Treatment

In order to determine the temperature at which the onset of gonadal sex differentiation occurs, juvenile scallops aged 6–12 months were collected every month from the Dalian Zhangzidao Fishery Group Corporation (Liaoning Province, China) from August 2018 to February 2019. The seawater temperature was recorded each time. After being transported to the laboratory, the scallops were acclimated in filtered and aerated seawater for 1 week at the temperature they were collected, varying from 3°C (in January and February) to 22°C (in August). For each month, about 30 healthy scallops were dissected, and their gonads were immediately frozen in liquid nitrogen and stored at −80°C for RNA extraction.

To determine the effect of temperature on gonadal sex differentiation, approximately 200 6 month-old sexually undifferentiated scallops were collected from the Yantai Beihuangdao Fishery Group Corporation (Shandong Province, China) and acclimated in filtered and aerated seawater for 3 days at the temperature they were collected (21°C). The scallops were then randomly divided into three groups (60 in each group) for temperature treatment (21, 16 and 11°C). For the 21°C group, the scallops were maintained at 21°C throughout the whole process. To prevent stimuli caused by rapid cooling, the latter two groups of scallops were placed in seawater with a decrease of temperature from 21°C by 0.5°C (16°C group) and 1°C (11°C group) every day for 10 days, respectively. About 20–30 individuals were sacrificed on day 0 and 50, and their gonads were dissected. The gonad mass was measured and gonadal index (GI) was calculated using gonad weight/total weight including the shell. Some of the gonads (10 gonads on day 0 and 15–20 on day 50) were immediately frozen in liquid nitrogen and stored at -80°C for RNA isolation. The remaining 10 gonads were fixed in 4% paraformaldehyde overnight, dehydrated with serial methanol (25, 50, 75 and 100%) diluted in 0.01 M phosphate-buffered saline and stored at −20°C for paraffin sectioning.

### Histological Analysis

After being dehydrated in ethanol, the samples were cleared in xylene, embedded in paraffin and cut into 5 μm-thick sections. The sections were placed on slides, dewaxed and hydrated, followed by staining with hematoxylin and eosin. Finally, the sections were observed and photographed using a Nikon’s Eclipse E600 research microscope.

### RNA Extraction

Total RNA was isolated using the TRIzol reagent according to the manufacturer’s instructions (Invitrogen, CA, United States). Potential DNA contamination was removed with DNase I (TaKaRa, Shiga, Japan) during the process. Purified RNA was quantified with Nanovue Plus spectrophotometer (GE Healthcare, NJ, United States) and the quality was assessed by agarose gel electrophoresis.

### Reverse Transcription Quantitative PCR (RT-qPCR)

To determine whether the gonads are sexually differentiated, relative expression of *FoxL2* and *Dmrt1L* was measured using RT-qPCR as described by Li et al. (2018). Briefly, first-strand cDNA was synthesized from 1 μg total RNA using oligo (dT)_18_ and MMLV reverse transcriptase (TaKaRa, Shiga, Japan). Each qPCR reaction contained 2 μl 5-fold diluted cDNA, 10 μl Light Cycler 480 SYBR Green I Master and 4 or 2 μM primers (Table 1). All reactions were conducted in triplicate, and performed on a Light Cycler 480 Real-time PCR System (Roche Diagnostics, Mannheim, Germany) using the following program: 94 °C for 10 min, and 40 cycles of 94°C for 15 s and 60°C for 1 min. In order for an easy comparison of LOG10 (*Dmrt1L*/*FoxL2*) values with Li et al. (2018) to discriminate sexually differentiated from undifferentiated samples, the calibrator sample of our previous study (Li et al., 2018) was used, which is mixed gonads from early differentiated 7-month-old ovaries. The expression of *FoxL2* and *Dmrt1L* was normalized to that of elongation factor 1-alpha (EF1A) using the 2^−ΔΔCt^ method.

**TABLE 1:** Sequences of the primers used for RT-qPCR assay.

Gene name	Primer sequences (5′-3′)	Amplicon length (bp)	References
FoxL2	F: AAC​TTC​TGG​ACA​TTG​GAC​CCT​GCT​T	134	Li et al. (2018)
	R: CCG​CAG​TGG​TTG​TCA​GCA​AAT​AAG​G		
Dmrt1L	F: ACA​GAT​TCC​CTA​CAG​ATG​CT	128	Li et al. (2018)
	R: TTA​TTC​ATG​GCG​GCG​TCT​AT		
EF1A	F: CCA​TCT​GCT​CTG​ACA​ACT​GA	196	Li et al. (2018)
	R: GGA​CAA​TAA​CCT​GAG​CCA​TAA		

### Statistical Analysis

To compare the differences in gonad weight, GI and relative expression levels of *FoxL2* and *Dmrt1L* between different groups, one-way ANOVA followed by LSD test was performed. SPSS25.0 was used for the analysis and *p*-values lower than 0.05 were considered statistically significant. The results were graphed using GraphPad8.0.

## Results

### Relationship Between Sex Differentiation Ratio and Seawater Temperature

**FIGURE 1:**
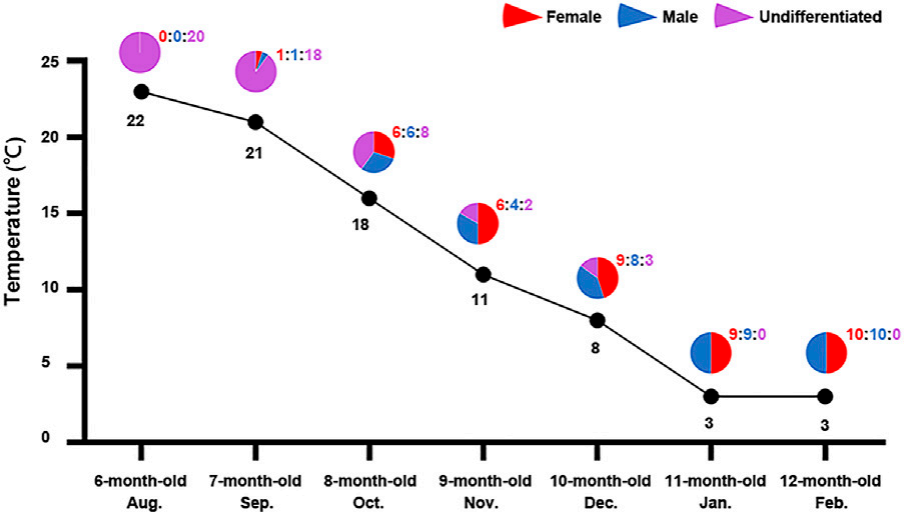
Sex differentiation ratio of 6–12 month-old scallops and the corresponding seawater temperature in each month. For each month, 12–20 individuals were examined. The pie charts show the percentages of female (red), male (blue) and undifferentiated samples (purple) in each month. The values above the pie charts are the corresponding numbers of female (red), male (blue) and undifferentiated samples (purple). The line indicates the seawater temperatures for each month.

In order to determine the effect of seawater temperature on sex differentiation of the Yesso scallop, we first examined the relationship between sex differentiation ratio and seawater temperature. Considering that molecular sex differentiation occurs earlier than morphological sex differentiation and it can be easily determined using relative expression of the female-biased gene *FoxL2* and male-biased gene *Dmrt1L* (Li et al., 2018), we tracked the sex differentiation rates of 6- to 12-month-old juvenile scallops for seven consecutive months in 2018–2019. As shown in Figure 1, all scallops were sexually undifferentiated at 6 months old in August (22°C), and sex differentiation started (10%) in 7 month-old scallops in September when the seawater temperature began to drop (21°C). One month later, 60% of the investigated individuals were sexually differentiated. All the scallops completed sex differentiation at 11 months old in January when seawater temperature dropped down to 3°C. The sex ratio was close or equal to 1:1 during the whole process. The sex differentiation rates in 2018–2019 were largely consistent with the results of our previous study in 2015–2016 (Li et al., 2018). According to the results, the increase in sex differentiation ratio corresponds to the decrease in seawater temperature, suggesting a potential effect of seawater temperature on gonadal sex differentiation of Yesso scallop.

### Morphological Changes in Gonads After Temperature Treatment

**FIGURE 2:**
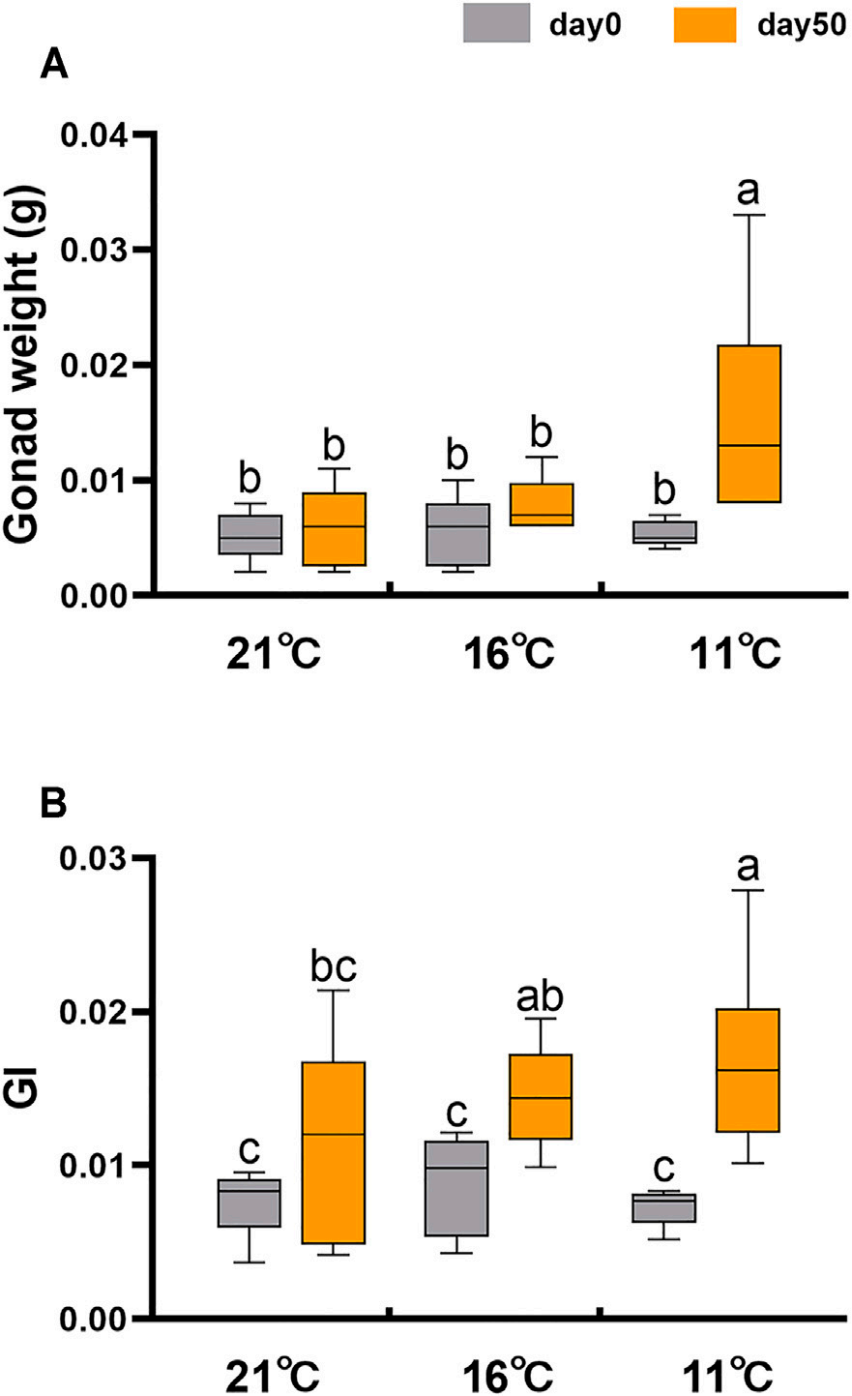
Gonad weight and gonadal index (GI) of juvenile scallops on day 0 and 50 at three different temperatures (21, 16 and 11°C). The vertical bars represent the means ± SEM (N = 5–6). One-way ANOVA followed by LSD test was performed to compare the differences among groups. Different letters indicate significant differences (*p* < 0.05).

To eliminate the effect of factors other than temperature on sex differentiation, we compared the gonadal development of sexually undifferentiated 6-month-old juvenile scallops at different temperatures under laboratory conditions. Considering that the thermal limit of Yesso scallop is ∼22°C (Xing et al., 2016), and the gonadal sex differentiation occurs during the decrease of seawater temperature, we set three temperature groups: 21, 16 and 11°C. As shown in Figure 2A, gonad weight was significantly increased for the 11°C group after 50 days of treatment, but no significant difference was found for the 21 or 16°C group. Gonad indices were increased for all three groups after 50 days of treatment (Figure 2B). However, changes were relatively smaller for the 21 and 16°C groups in contrast to the 11°C group. These results indicate that low temperature accelerates gonadal growth.

**FIGURE 3:**
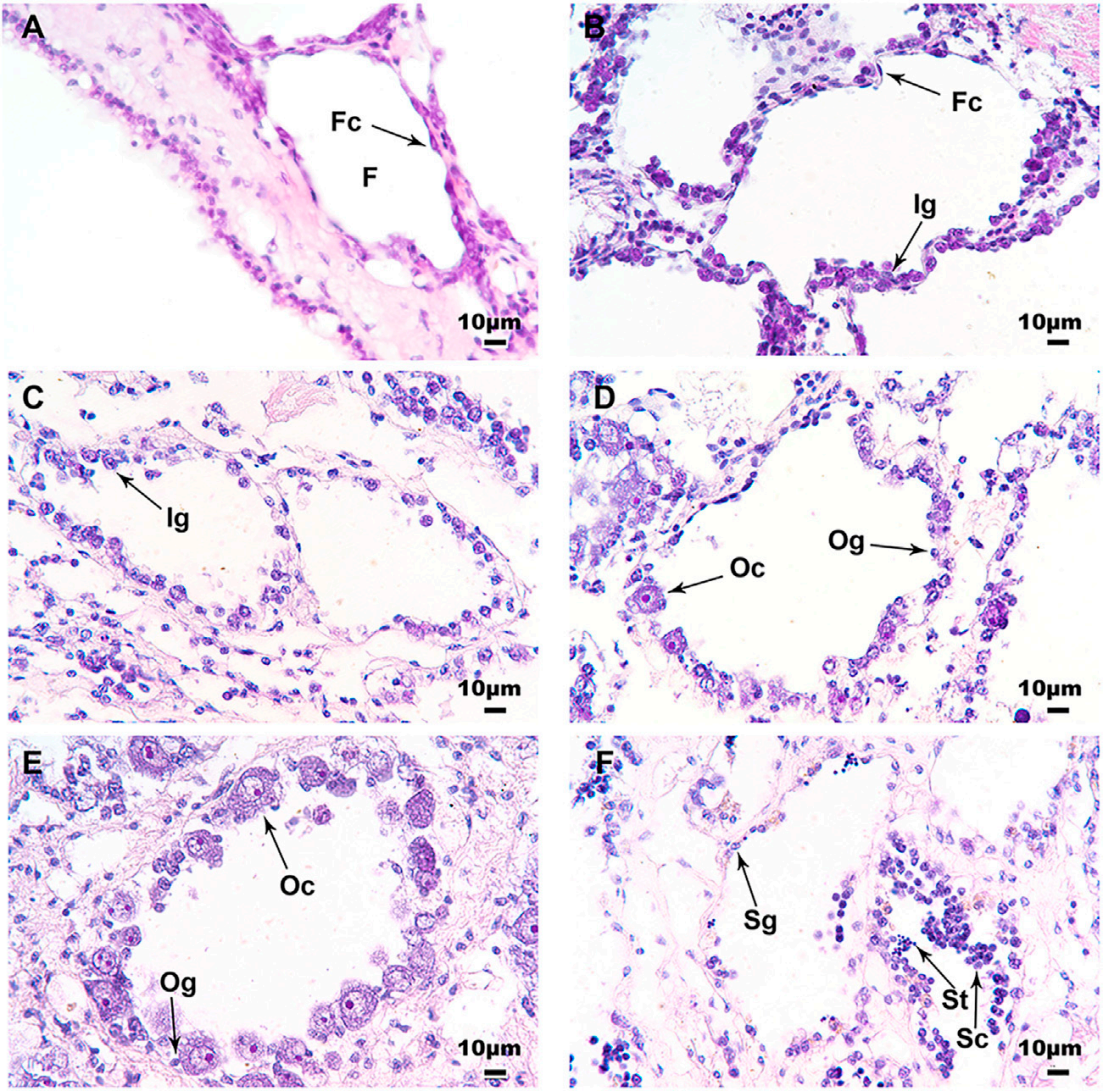
Histological observations of the juvenile gonads on day 0 and 50 at three different temperatures. **(A)** Undifferentiated gonad of a 6 month-old juvenile scallop on day 0; **(B)** Gonad from the 21°C group on day 50; **(C)** Undifferentiated gonad from the 16°C group on day 50; **(D)** Ovary from the 16°C group on day 50; **(E)** Ovary from the 11°C group on day 50; **(F)** Testis from the 11°C group on day 50. Six scallops were sampled for histological analysis at each stage. F, follicle; Fc, follicle cell; Ig, indistinguishable gonium; Og, oogonium; Oc, oocyte; Sg, spermatogonium; Sc, spermatocyte; St, spermatid.

The histological analysis confirmed the influence of temperature on the development of scallop gonads. Figure 3A displays the gonad of 6 month-old juveniles at day 0, in which the follicles were generally empty, with a monolayer of follicle cells surrounding the inner wall. After 50 days of treatment at 21°C, more cells can be observed in the follicles, including some sexually indistinguishable gonia (Figure 3B). For the 16°C group, most of the samples displayed similar morphology with the 21°C group at day 50 (Figure 3C), but a small number of them were sexually distinguishable, with some oocytes or spermatocytes scattering in the follicles (Figure 3D). Most of the samples from the 11°C group were sexually differentiated, with various types of germ cells being observed, including oocytes in the ovary (Figure 3E) or spermatocytes and spermatids in the testis (Figure 3F).

### Effect of Temperature on the Expression of *FoxL2* and *Dmrt1L* in the Gonads

**FIGURE 4:**
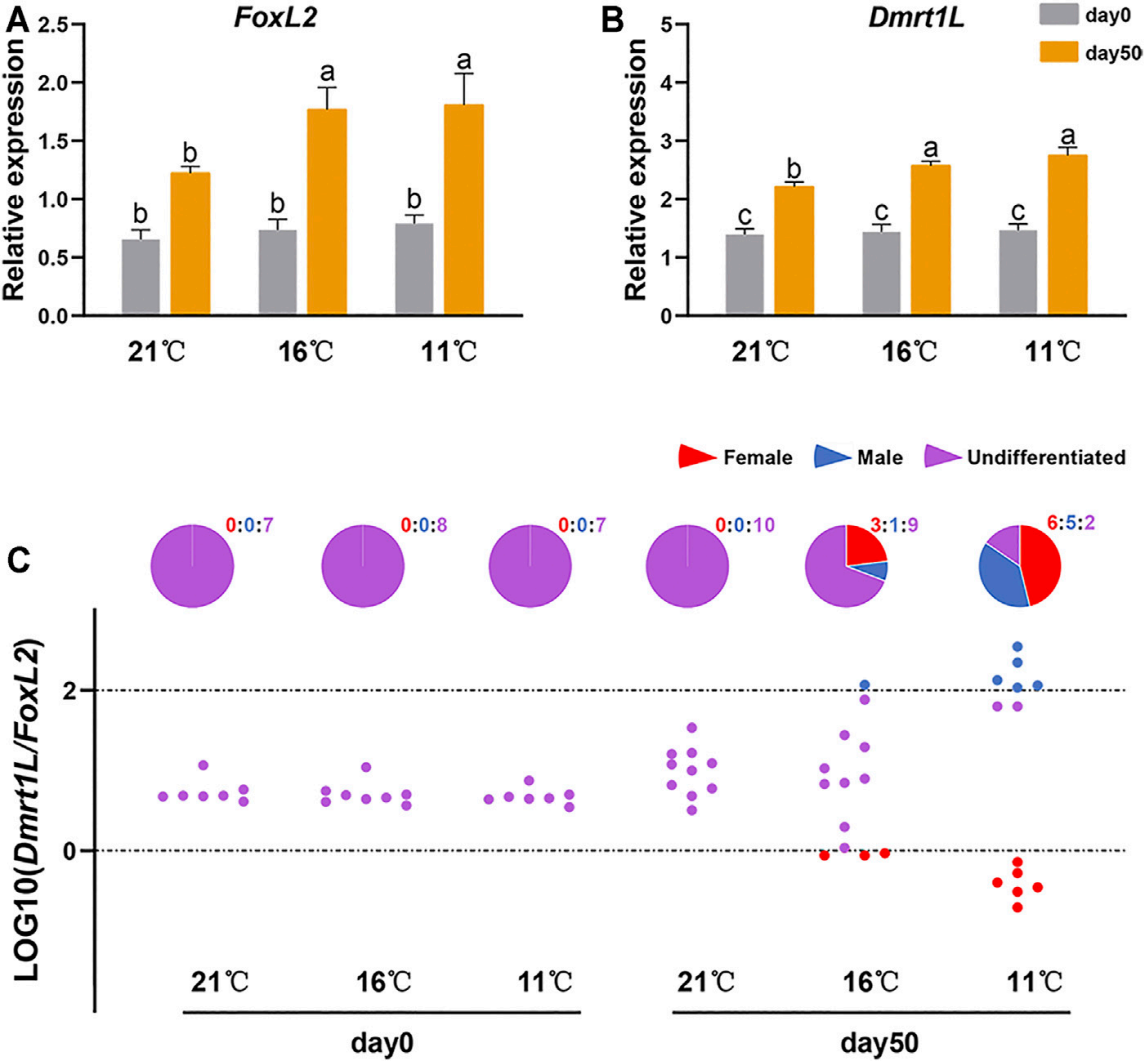
Relative expression of *FoxL2* and *Dmrt1L* in the three temperature groups at day 0 and 50. Relative expression levels of *FoxL2* **(A)** and *Dmrt1L* **(B)** in the three temperature groups (21, 16 and 11°C) at day 0 and 50. The vertical bars represent the means ± SEM (N = 7–13). Different letters indicate significant differences (*p* < 0.05). **(C)** The LOG10 (*Dmrt1L*/*FoxL2*) values and sex differentiation ratio for each group on day 0 and 50. Each dot represents a sample. The dashed lines indicate the threshold for sex differentiation, with values below 0 for ovary and values higher than two for testis. The pie charts indicate the percentages of female (red), male (blue) and undifferentiated samples (purple) in each month. The values above the pie charts are the corresponding numbers of female (red), male (blue) and undifferentiated samples (purple).

We further examined the relative expression levels of *FoxL2* and *Dmrt1L* in the gonads of the three temperature groups. According to the results, the expression levels of *FoxL2* (Figure 4A) and *Dmrt1L* (Figure 4B) were elevated after 50 days of treatment for all three groups. On day 50, both genes were significantly higher in the 11 and 16°C groups compared to the 21°C group. Considering that *FoxL2* and *Dmrt1L* are distributed in germ cells and follicle cells in scallops (Li et al., 2018; Liu et al., 2012; Wei et al., 2021), the results indicate that proportion of these two types of cells increased after 50 days of treatment, especially for the 11°C and 16°C groups, consistent with the histological observations (Figure 3).

LOG10 (*Dmrt1L*/*FoxL2*) was used to distinguish differentiated from undifferentiated samples, with values below 0 for ovary and values higher than two for testis (Li et al., 2018). According to the standard, all the day 0 samples were sexually undifferentiated. However, sex differentiation rates varied between groups on day 50, with 0 (0/10) for the 21°C group, 30.77% (4/13) for the 16°C group and 84.62% (11/13) for the 11°C group (Figure 4C). Of the 15 differentiated samples, six were testes and nine were ovaries. The sex ratio of the 11°C group at day 50 was 0.83:1, close to 1:1.

## Discussion

Temperature is well known to be an external cue influencing gonadal differentiation in many poikilotherms. However, the effects can be different between species, varying from changing the speed of gonadal development to altering sex ratio (Hayashi et al., 2010; Pankhurst and King, 2010; Nivelle et al., 2019; Yu et al., 2021). Our study demonstrates from the morphological and molecular way that temperature doesn’t seem to alter sex ratio in the Yesso scallop because both males and females were observed in the 16 and 11°C group on day 50. However, temperature can influence the speed of gonadal development in Yesso scallop. Based on histological analysis, the morphology of 21 and 11°C groups on day 50 resembles that of 8 and 10-month-old juveniles from natural population, respectively (Li et al., 2018). The molecular sex differentiation rates of 21°C (0%) and 11°C groups (84.62%) on day 50 are close to those of 6 (0%) and 10-month-old (85%) scallops under natural conditions (Figure 1). These results suggest that in the laboratory, 11°C treatment accelerates sex differentiation and gametogenesis of Yesso scallop by approximately 2 months in contrast to the natural population, possibly because Yesso scallop is a cold-water species with an optimal growth temperature of 4–8°C (FAO, 2013).

Not all organisms exhibit the same response to temperature treatment. It varies widely between species regarding whether temperature treatments promote or inhibit gonadal development. Similar with Yesso scallop, gametogenesis is more rapid at low temperatures and slowed at high temperatures in mussel *Mytilus galloprovincialis* (Fearman and Moltschaniwskyj, 2010). However, it is quite different in other bivalves. For example, high temperature results in an increase of gonadal index in the mussel *Modiolus barbatus* (Mladineo et al., 2007), and accelerates gametogenesis in the Pacific oyster *C. gigas* (Fabioux et al., 2005), rock oyster *Striostrea prismatica* (Argüello-Guevara et al., 2013) and mussel *Mytilopsis leucophaeata* (Verween et al., 2009). Therefore, we assume that in bivalves, seawater temperature could play a critical role in sex differentiation and gametogenesis, but it varies among species in terms of which kind of variation (i.e. increase or decrease) accelerates this process.

An interesting phenomenon is that treatment at high temperature (21°C) entirely inhibits sex differentiation of Yesso scallop. This group of scallops have fewer germ cells in the follicle, and lower levels of *FoxL2* and *Dmrt1L* than the 11°C group. Considering that 21°C is close to the thermal limit (22°C) of Yesso scallop (Xing et al., 2016), the long-term treatment at 21°C may reduce the expression of key sex differentiation genes, thereby inhibiting the cell differentiation process. Actually, high temperature (28°C) also delays gonadal differentiation in the oyster *C. gigas* (Santerre et al., 2013), and in some fish, above-normal temperatures are demonstrated to have deleterious effects on reproductive processes (Pankhurst and King, 2010). The thermal inhibition of vitellogenesis has been proposed to be associated with suppress of a target of FoxL2, the aromatase, which mediates the conversion of testosterone to estradiol (Pankhurst and King, 2010). Therefore, we speculate that juvenile scallops can perceive changes in seawater temperature, deliver it to the gonads, and induce or suppress the expression of sex differentiation genes (e.g. *FoxL2*, *Dmrt1L*), thereby accelerating or inhibiting morphological sex differentiation. The exact mechanism about how this process takes place warrants further investigation.

There is a small discrepancy in the effect of temperature on sex differentiation between populations in the laboratory and field. In the laboratory, sex differentiation does not occur at 21°C. But under natural conditions, we observed a sex differentiation rate of 10% in 7 month-old scallops collected at 21°C. This can be explained by the following reasons. First, there is fluctuation in seawater temperature. Therefore, it is possible that the scallops have gone through a temperature lower than 21°C before they were collected. Second, it is well documented that diet could affect gonadal development (Teaniniuraitemoana et al., 2016). In our case, it is likely that nutritional status at sea is better than that of the laboratory, thus the natural population could develop faster than the laboratory one. Besides, natural photoperiod, the presence of minerals, metals, and other compounds in the water, may also contribute to this discrepancy.

Juvenile Yesso scallop has long been regarded as protandrous hermaphrodite that first differentiate into males and undergo sex reversal to female thereafter (Mori et al., 1977; Maru, 1978; Otani et al., 2017). However, there is no evidence as yet for existence of this male to female conversion process. In our study, both females and males were observed in early differentiated scallops, and no obvious sex bias was found during the developmental process. Therefore, we speculate that juvenile Yesso scallop is gonochoristic, just like adults, rather than protandrous hermaphrodite.

## Conclusion

In summary, we demonstrated that temperature can influence gonadal sex differentiation in Yesso scallop. In the field, sex differentiation occurs in September, when the temperature begins to drop. Under laboratory conditions, we found 21°C inhibits sex differentiation, whereas 11°C accelerates sex differentiation by 2 months. Our study also supports that juvenile Yesso scallop has a gonochoristic sexual system rather than protandrous hermaphroditism. It addresses for the first time an environmental influence associated with genetic controls on scallop sex differentiation.

## Data Availability

The original contributions presented in the study are included in the article/supplementary material, further inquiries can be directed to the corresponding author.
